# Epigenetic Profiling of Type 2 Diabetes Mellitus: An Epigenome-Wide Association Study of DNA Methylation in the Korean Genome and Epidemiology Study

**DOI:** 10.3390/genes14122207

**Published:** 2023-12-13

**Authors:** Hyein Seo, Jae-Ho Park, Jin-Taek Hwang, Hyo-Kyoung Choi, Soo-Hyun Park, Jangho Lee

**Affiliations:** Korea Food Research Institute, Wanju-gun 55365, Jeollabuk-do, Republic of Korea; hiseo@kfri.re.kr (H.S.); jaehopark@kfri.re.kr (J.-H.P.); jthwang@kfri.re.kr (J.-T.H.); chkyoung@kfri.re.kr (H.-K.C.); shpark0204@kfri.re.kr (S.-H.P.)

**Keywords:** type 2 diabetes mellitus, KoGES, epigenetic landscape, DNA methylation, EWAS

## Abstract

Diabetes is characterized by persistently high blood glucose levels and severe complications and affects millions of people worldwide. In this study, we explored the epigenetic landscape of diabetes using data from the Korean Genome and Epidemiology Study (KoGES), specifically the Ansung–Ansan (AS–AS) cohort. Using epigenome-wide association studies, we investigated DNA methylation patterns in patients with type 2 diabetes mellitus (T2DM) and those with normal glucose regulation. Differential methylation analysis revealed 106 differentially methylated probes (DMPs), with the 10 top DMPs prominently associated with TXNIP, PDK4, NBPF20, ARRDC4, UFM1, PFKFB2, C7orf50, and ABCG1, indicating significant changes in methylation. Correlation analysis highlighted the association between the leading DMPs (e.g., cg19693031 and cg26974062 for TXNIP and cg26823705 for NBPF20) and key glycemic markers (fasting plasma glucose and hemoglobin A1c), confirming their relevance in T2DM. Moreover, we identified 62 significantly differentially methylated regions (DMRs) spanning 61 genes. A DMR associated with PDE1C showed hypermethylation, whereas DMRs associated with DIP2C, FLJ90757, PRSS50, and TDRD9 showed hypomethylation. PDE1C and TDRD9 showed a strong positive correlation between the CpG sites included in each DMR, which have previously been implicated in T2DM-related processes. This study contributes to the understanding of epigenetic modifications in T2DM. These valuable insights can be utilized in identifying potential biomarkers and therapeutic targets for effective management and prevention of diabetes.

## 1. Introduction

Diabetes is a chronic disease that affects the ability of the body to produce or utilize insulin, which is necessary for regulating blood sugar levels. Over time, high blood sugar levels can lead to serious, life-threatening complications that can affect different body parts, including the eyes, kidneys, nerves, and cardiovascular system [1]. The International Diabetes Federation has reported that approximately 537 million adults aged between 20–79 years were affected by diabetes in 2021, accounting for approximately 9.3% of the total global population. The prevalence of diabetes is increasing at a significant pace, and the number of people with diabetes will reach 700 million by 2045 [2].

An epigenome-wide association study (EWAS) is a study that aims to identify epigenetic changes associated with a particular trait or disease. Epigenetic changes refer to the modifications of DNA and associated proteins that do not involve changes in the underlying genetic code [3]. EWAS has several applications, including identification of epigenetic changes associated with complex diseases and environmental exposure, discovery of epigenetic biomarkers, identification of potential therapeutic targets, and understanding the mechanisms underlying complex diseases. A genome-wide association study (GWAS), which is a study that tests the associations of a plethora of single nucleotide polymorphisms (SNPs) that encompass the entire genome with a specific trait [4], has been applied to identify genetic risk factors for T2DM, and, to date, more than 120 genetic variants have been found to be associated with T2DM risk [5]. However, epigenomic profiling studies have been less performed for T2DM in humans.

An EWAS strives to identify epigenomic variants associated with a phenotype of interest, which provides complementary information to a GWAS. So far, most EWASs have applied DNA methylation microarrays such as the Illumina Infinium HumanMethylation BeadChip arrays. Although genomic variations are static, epigenomic variations tend to be dynamic, and interindividual epigenetic variabilities could play a critical role in disease pathogenesis. Rather than studying rare changes in the epigenome, currently, an EWAS mainly studies common DNA methylation variations in the population, which could be a powerful approach in unraveling risk-associated epigenetic biomarkers [6]. Several EWASs have identified epigenetic changes associated with both type 1 and type 2 diabetes mellitus and complications such as diabetic nephropathy and retinopathy. Furthermore, the potential of EWAS has been leveraged to investigate the impact of environmental factors on the risk of having diabetes and explore potential targets of intervention for preventing and treating diabetes [7,8].

EWASs have been used in several cohorts to identify epigenetic changes associated with diabetes. For instance, an EWAS conducted in the EPIC-Norfolk Study has identified changes in DNA methylation associated with T2DM, which were linked to the expression of genes involved in insulin signaling and glucose metabolism [9]. In addition, a meta-analysis of four European cohorts has identified 227 differentially methylated positions associated with T2DM, many of which were located in genes involved in glucose metabolism and insulin signaling pathways [10].

The Korean Genome and Epidemiology Study (KoGES) is a large-scale and population-based investigation aimed at exploring the genetic and environmental factors underlying common diseases in the Korean population. KoGES has generated an extensive dataset on DNA methylation from the Ansung–Ansan (AS–AS) cohort, which is valuable for exploring the impact of epigenetic modifications on various health outcomes [11]. Ko et al. (2022) examined the correlation between fatty liver index (FLI) and DNA methylation patterns in the Korean population using the Illumina Infinium HumanMethylation 450k (HM450k) data and observed that FLI is linked to alterations in DNA methylation of genes involved in lipid metabolism, inflammation, and insulin resistance [12]. Kim et al. (2023) have demonstrated an association between DNA methylation patterns across approximately 400,000 CpG sites and development of chronic kidney disease in KoGES [13]. Although the AS–AS cohort within KoGES encompasses the Illumina Infinium HumanMethylation 850k (HM850k) data for more than 1000 participants, HM450k has been commonly utilized for EWAS in this cohort.

In this study, we examined the distinct methylation patterns between participants with T2DM and those with normal glucose regulation using the extensive HM850k dataset from the AS–AS cohort to identify DMPs and assessed their correlation with key blood markers of diabetes, including fasting plasma glucose (FPG) and hemoglobin A1c (HbA1c). Additionally, we identified DMRs in individuals with T2DM compared to those in individuals with normal glucose regulation and conducted further analyses to explore co-methylation patterns.

## 2. Materials and Methods

### 2.1. Participants and Data Source

Clinical, epidemiological, and DNA methylation array datasets were obtained from the AS–AS cohort of the KoGES, facilitated by the Korea Center for Disease Control and Prevention [11]. Participants’ ages ranged between 40 and 69 years. For this study, data from the HM850k array were utilized, specifically from the 5th follow-up cohort, which encompassed 1528 samples with 865,918 CpG probes. This study was ethically approved by the Institutional Review Board (IRB) of the Korea Food Research Institute (Approval No.: 2022-01-002-001).

### 2.2. Study Design

**Figure 1:**
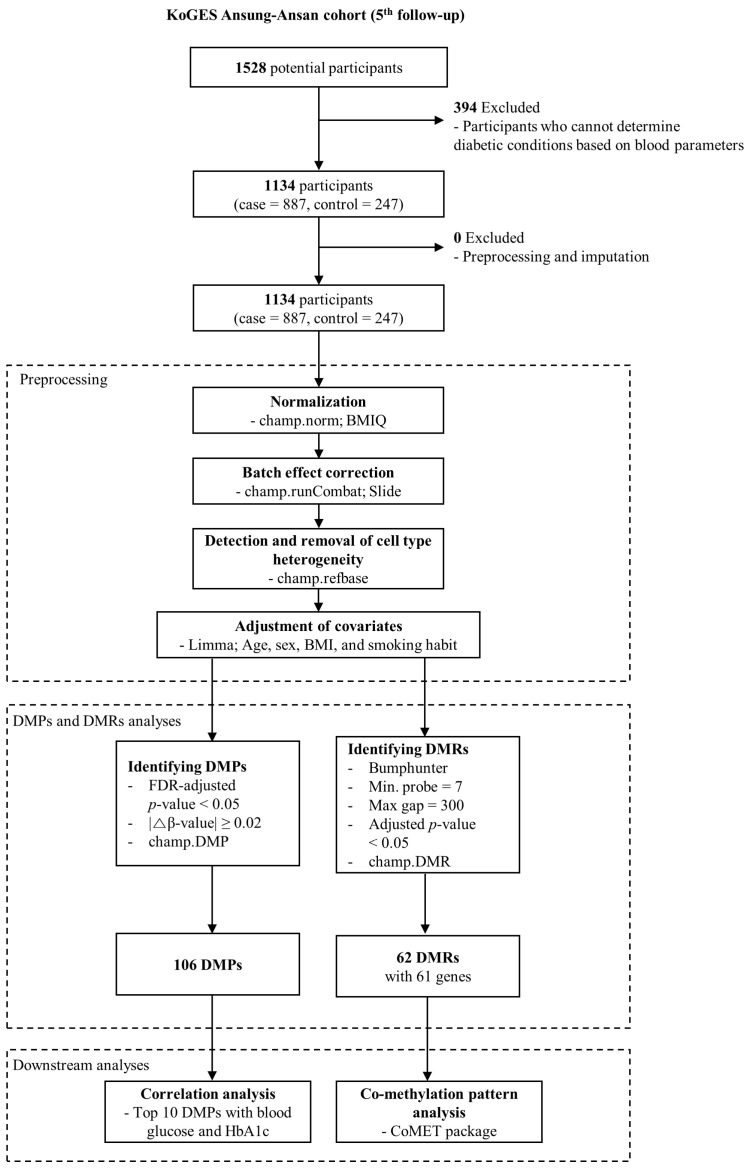
Flowchart of this study. Among the initial 1528 potential participants from the AS-AS cohort in KoGES at the 5th follow-up, individuals were categorized into either the T2DM (case) or normal (control) groups based on blood parameters, including FPG, HbA1c, and 2 h PG, as described in the Section 2. This process resulted in 1134 remaining participants (T2DM = 887, normal = 247) eligible for DNA methylation analysis. Subsequently, the HM850k data for these 1134 samples underwent preprocessing, involving normalization using the β-mixture quantile normalization (BMIQ) method, technical batch effect correction for slides using the Combat algorithm, and adjustment for covariates (utilizing the limma package; confounders = age, sex, BMI, and smoking habit), and removal of cell type heterogeneity using the champ.refbase function. The β-value matrix resulting from the preprocessing of raw idat files was then employed for analyses of DMPs and DMRs. The identified DMPs were subjected to correlation analysis with FPG or HbA1c. Additionally, selected DMRs from the pool of identified DMRs underwent co-methylation pattern analysis using the CoMET Bioconductor package.

The study design is depicted in Figure 1. T2DM cases were identified based on abnormal levels of FPG and HbA1c and plasma glucose level after 2 h (2 h PG) levels. Specifically, abnormalities were defined as FPG level ≥ 126 mg/dL, HbA1c level ≥ 6.5%, or 2 h PG ≥ 200 mg/dL. For an individual to be classified as having T2DM, two or more of these conditions needed to be simultaneously fulfilled [1]. Individuals with undetermined T2DM or normal status were excluded from the analysis. After processing the data of DNA methylation for normalization, correction for technical batch effects, adjustment for covariates (age, sex, body mass index (BMI), and smoking habit), and corrections of cell type heterogeneity, DMPs, and DMRs were identified. We selected the 10 top DMPs and subjected them to correlation analyses with FPG and HbA1c levels (*p* < 0.05; Spearman correlation). Additionally, we focused on DMRs that exhibited significant CpG probes numbering over three and performed an in-depth analysis of their co-methylation patterns using the “CoMET” package v.1.34.0 in Bioconductor [14].

### 2.3. Data Preprocessing and Adjustment of Confounding Effects

The HM850k data for a cohort of 1134 participants (887 with T2DM and 247 classified as normal) were imported using champ.load function of the “ChAMP” Bioconductor package v.2.26.0 [15]. Following this, a filtration process (Supplementary Figure S1) resulted in the selection of 723,301 CpG probes from an initial pool of 865,918 probes. Discrepancies between the two types of probes, probe I and probe II, were addressed through the BMIQ method [16], using the champ.norm function. To counteract batch effects stemming from different slides, the champ.SVD function was used for detection of Principal Components, the components correlated with the covariates, followed by correction using champ.runcombat function, both integral to the ChAMP package (Supplementary Figure S2). Additionally, confounding factors, specifically age, sex, BMI, and smoking habit were accounted for by adjustments using the limma Bioconductor package [17]. The Q-Q plot, assessing the model fit, is presented in Supplementary Figure S3.

### 2.4. Identification of DMPs

The DMPs distinguishing individuals with T2DM from normal individuals were identified using champ.dmp function within the ChAMP Bioconductor package. To qualify as DMPs, the CpG probes had to satisfy two criteria: a false discovery rate (FDR) also called a multiplicity-adjusted *p*-value < 0.05 and an absolute β-value ≥ 0.02. The DMPs meeting these criteria were graphically represented using a volcano plot employing EnhancedVolcano Bioconductor package for visualization [18].

### 2.5. Identification of DMRs

The DMRs were identified using champ.dmr function from ChAMP package, and Bumphunter algorithm with maximum gap of 300 bp, minimum seven probes, and an adjusted *p*-value < 0.05 was used for identifying DMRs.

### 2.6. Statistical Analyses

Statistical analyses were performed using R v.4.2.1. Student’s *t*-test was employed to determine the significant differences in means of β-values between two groups (*p* < 0.05). Furthermore, the correlation coefficient (*R*) between β-values of the identified DMPs and blood parameters, such as FPG or HbA1c, was calculated using Spearman’s correlation analysis (*p* < 0.05).

## 3. Results

### 3.1. Clinical Characteristics of the Study Participants

In the KoGES cohort study, the participants with normal glycemic status (*n* = 247) and those with T2DM (*n* = 887) were compared. Fasting glucose levels were significantly higher in the T2DM group (161.05 mg/dL) than in the normal group (89.26 mg/dL). Similarly, HbA1c levels were elevated in participants with T2DM (7.56%) compared to those in the normal group (5.35%). The 2 h PG levels were higher in participants with T2DM (261.96 mg/dL) than in those in the normal group (105.49 mg/dL). Additionally, individuals with T2DM had a higher mean BMI (25.31 kg/m^2^) than had the normal individuals (23.64 kg/m^2^). The normal group comprised 50.20% males, whereas the T2DM group comprised 57.50% males. The mean age of participants in the normal group was 57.77 years (standard deviation (SD), 8.38), whereas that in the T2DM group was 62.45 years (SD, 8.22). The difference in the mean age between the two groups was statistically significant (*p* < 0.001). Additionally, the prevalence of smoking was higher in the T2DM group (47.30%) compared to the normal group (37.80%) (Table 1). These findings underscore the significant impact of T2DM on glycemic control and associated metabolic parameters while highlighting age, sex, BMI, and smoking habit as potential confounders within the KoGES cohort.

### 3.2. Identification of DMPs

**Figure 2:**
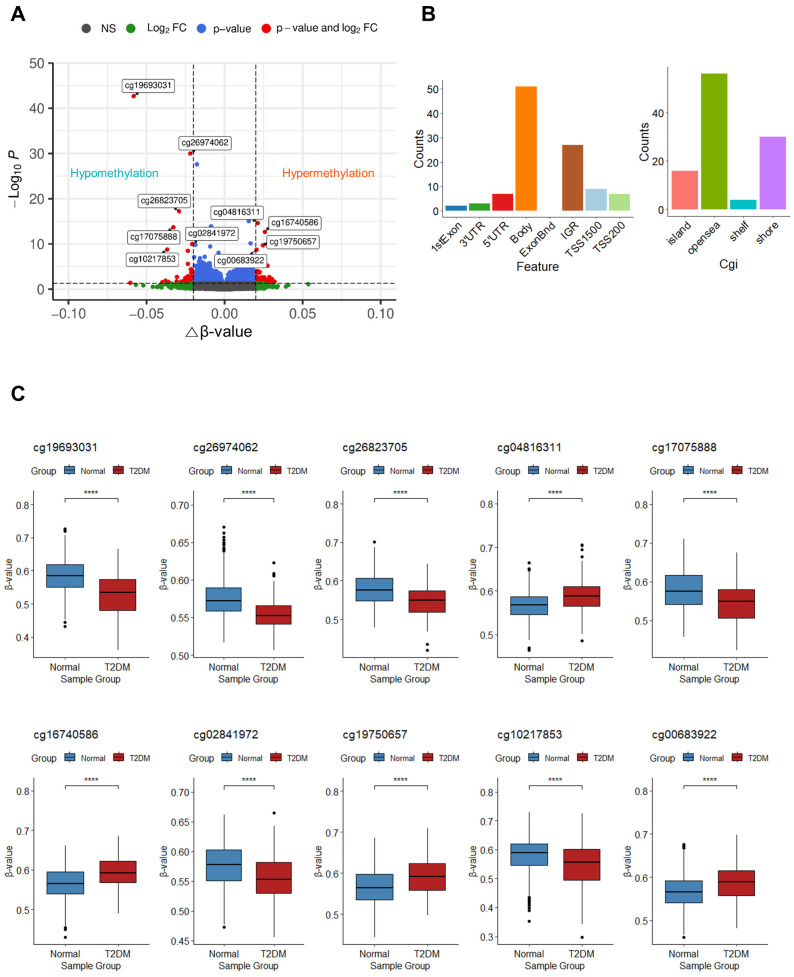
DMP analysis results. (**A**) Volcano plot of the DMPs. The 10 top DMPs are labeled with the names of their CpG probes, and all DMPs are depicted as red dots. The *x*-axis represents △β-values between T2DM and normal groups, whereas the *y*-axis indicates FDRs, also called multiplicity-adjusted *p*-values. A total of 723,301 CpG probes are depicted as dots. Dark grey, green, blue, and red dots correspond to not significant (n.s.), |△β-values| ≥ 0.02 and adjusted *p*-value ≥ 0.05, |△β-values| < 0.02 and adjusted *p*-value < 0.05, and |△β-values| ≥ 0.02 and adjusted *p*-value < 0.05, respectively. (**B**) Distribution of the locations of CpG sites in DMPs. The left-panel histogram displays the distribution of CpG sites in various regions, including the first exon, 3′ untranslated region (UTR), 5′UTR, gene body, intergenic region, transcription start site (TSS) 1500, and TSS200. The *y*-axis represents the count of each region. The right-panel histogram illustrates the distribution of CpG sites in different locations including CpG island, open sea, shelf, and shore. The *y*-axis represents the count of each CpG location. (**C**) Box plots for 10 top DMPs. The *x*-axis represents the sample groups, and the *y*-axis represents β-values for each group. Outliers are depicted as dots. Asterisks (****) indicate significant differences between the normal and diabetes groups (*p* < 0.0001; Student’s *t*-test).

To identify the features of DNA methylation associated with T2DM, we analyzed DMPs between the T2DM and normal groups. A total of 106 DMPs were detected with an adjusted *p*-value < 0.05 and an absolute change in β-value ≥ 0.02. Among these, 61 DMPs exhibited hypermethylation, whereas 45 DMPs displayed hypomethylation in patients with T2DM (Figure 2A). Within these 106 DMPs, CpG sites were predominantly enriched in the gene body (51 CpGs; 48.11%), followed by the intergenic region (IGR; 27 CpGs; 25.47%), transcription start site 1500 (TSS1500; 9 CpGs; 8.49%), 5′ untranslated region (5′UTR; 7 CpGs; 6.60%), transcription start site 200 (TSS200; 7 CpGs; 6.60%), 3′ untranslated region (3′UTR; 3 CpGs; 2.83%), and the first exon (2 CpGs; 1.89%) (Figure 2B, left panel). The majority of CpG sites were located in open sea regions (56 CpGs; 52.83%), followed by shore (30 CpGs; 28.30%), island (16 CpGs; 15.09%), and shelf (4 CpGs; 3.77%) (Figure 2B, right panel). The 10 top DMPs are documented in Table 2, with the two highest-ranked DMPs (cg19693031 and cg26974062) found within the 3′UTR or body of *TXNIP*. These two DMPs exhibited significant hypomethylation in patients with T2DM (*p* < 0.05). Similarly, cg17075888, cg26823705, and cg10217853 within *PDK4*, *NBPF20*, and *ARRDC4*, respectively, showed significant hypomethylation, whereas cg04816311, cg16740586, cg19750657, and cg00683922 located within *C7orf50*, *ABCG1*, *UFM1*, and *PFKFB2* displayed significant hypermethylation in patients with T2DM, respectively. Additionally, substantial hypomethylation of one of the 10 top DMPs, cg02841972, which are found in the intergenic region of chromosome 2, was noticed (*p* < 0.05) (Figure 2C).

### 3.3. Correlation between Methylation Levels of Top DMPs and Glycemic Markers (FPG and HbA1c)

**Figure 3:**
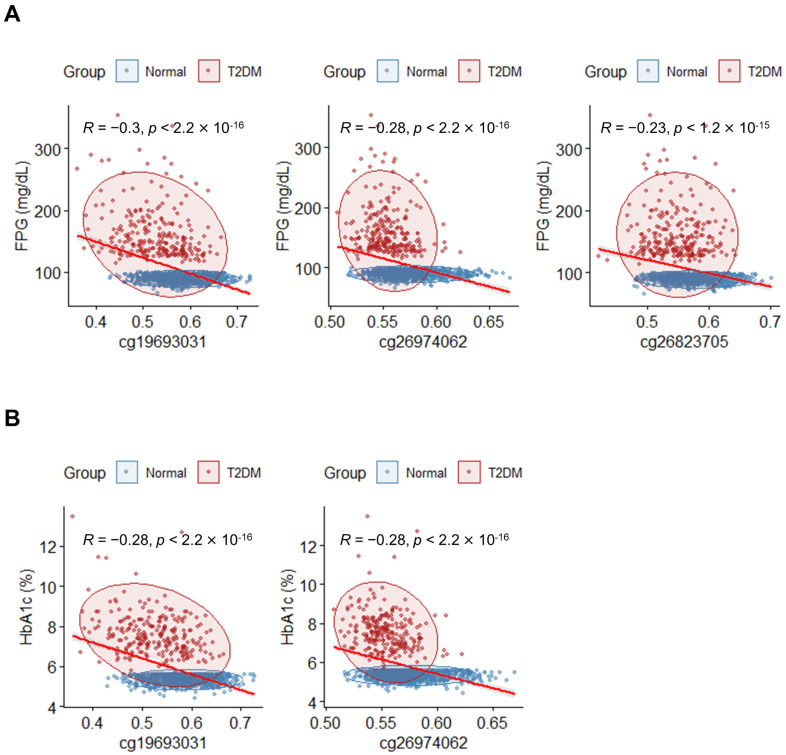
Correlation between methylation levels of DMPs and diabetic markers of the blood. (**A**) Correlation analysis between FPG and DMPs with *p* < 0.05; |R| ≥ 0.2 (Spearman correlation). The *x*-axis indicates β-values for individuals, and the *y*-axis indicates FPG levels (mg/dL). Individuals in the T2DM and normal groups are indicated by red and blue dots, respectively. The red line represents the regression line. (**B**) Correlation analysis between HbA1c and DMPs with *p* < 0.05; |R| ≥ 0.2 (Spearman correlation). The *x*-axis indicates β-values for individuals, and the *y*-axis indicates HbA1c (%). Individuals in the T2DM and normal groups are indicated by red and blue dots. The red line represents the regression line.

To validate the link between the previously identified DMPs and T2DM, a correlation analysis was performed between the methylation levels of the 10 top DMPs and markers associated with T2DM, including FPG and HbA1c. Spearman’s correlation coefficient (R) was calculated for each CpG probe and the diabetic markers. Three DMPs (cg19693031, cg26974062, and cg26823705) exhibited significant negative correlations (Figure 3A). For HbA1c, two DMPs (cg19693031 and cg26974062) demonstrated significant negative correlations (Figure 3B). Notably, the β-values of cg19693031 and cg26974062 were significantly correlated with FPG and HbA1c.

### 3.4. Identification of DMRs

**Figure 4:**
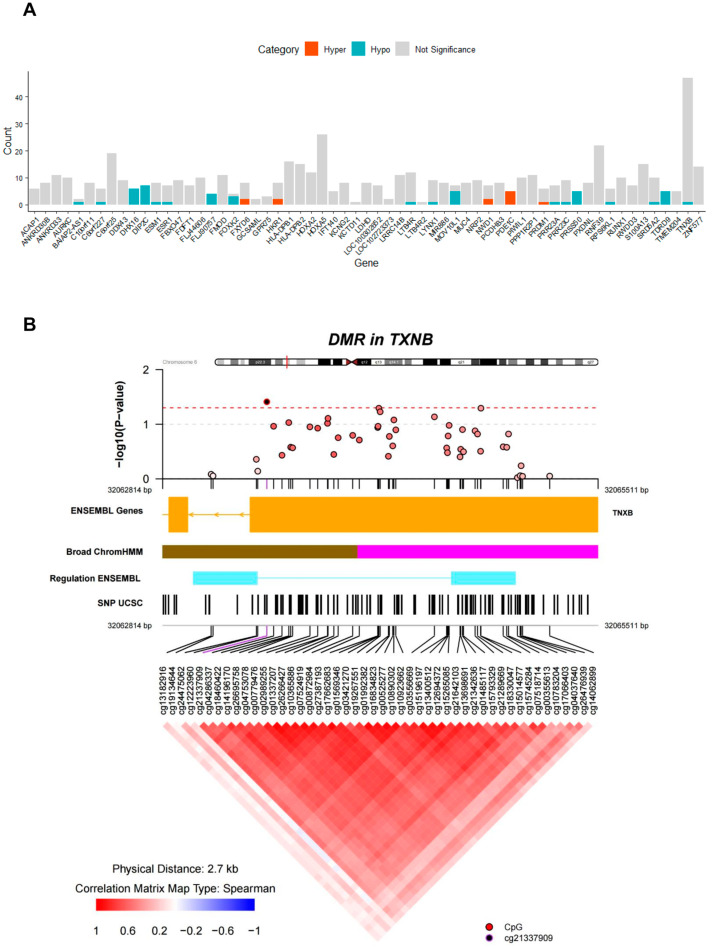
Results of DMR analysis. (**A**) Bar graph illustrating counts of hypermethylated or hypomethylated DMPs in each DMR. Dark orange, cyan, and light grey sections in the bar graph indicate the count of hypermethylated, hypomethylated, and not significant probes, respectively. (**B**) Analysis of co-methylation pattern for the DMR surrounding *TXNB*. The upper panel displays a regional Manhattan plot of the DMR surrounding *TXNB*. The *y*-axis represents −log_10_(*p*-value) for each CpG probe in the DMR, and the *x*-axis represents the genomic location of each CpG site. In the middle panel, annotation tracks for gene, broad ChromHMM, regulation, and SNPs are presented. Within the broad ChromHMM track, pink and brown tracks correspond to repressed and heterochromatin/low signal regions, respectively, and, within the regulation ENSEMBL track, sky blue track corresponds to CTCF binding site, as defined in the coMET user guide (https://www.bioconductor.org/packages/devel/bioc/vignettes/coMET/inst/doc/coMET.pdf; accessed on 30 November 2023). In the lower panel, the co-methylation pattern of CpG probes within the DMR is depicted, displaying a heatmap of the Spearman correlation coefficients (*R*). In this heatmap, red indicates a positive correlation with relatively high *R* values, whereas blue indicates a negative correlation with relatively low *R* values.

Subsequently, we identified the genomic regions exhibiting divergent patterns of DNA methylation between the diabetic and normal groups. We identified 62 significant DMRs (*p* < 0.05). Further enrichment analysis revealed the involvement of 61 genes in this process (Figure 4A). The 10 highest-ranked DMRs are listed in Table 3. Remarkably, DMR_1 situated within *TXNB* had the highest impact. Additionally, the 10 top DMRs encompassed annotations for genes such as *RNF39*, *MIR886*, *DIP2C*, *S100A13*, *HLA*-*DPB1*, and *C6orf25* (Table 3). Upon delving into co-methylation pattern analysis within *TXNB*, we observed a tendency towards positive correlation in the methylation levels of individual CpG sites within the genomic region containing the CpG island of *TXNB*. However, 47 out of the 48 CpG sites within the DMR for *TXNB* was not significant as a differentially methylated probe (adjusted *p*-value < 0.05) (Figure 4B). For DMRs related to *PDE1C*, more than 50% CpG sites within each DMR exhibited significant hypermethylation. Conversely, for *DIP2C*, *FLJ90757*, *PRSS50*, and *TDRD9*, we observed significant hypomethylation (adjusted *p*-value < 0.05) (Figure 4A). Furthermore, co-methylation pattern analysis of the DMRs for *PDE1C* and *TDRD9* showed a pronounced positive correlation among the CpG sites encompassed within each DMR, respectively (Supplementary Figures S4 and S5, respectively).

## 4. Discussion

In this study, we performed DNA methylome analysis of approximately 850,000 CpG sites from blood samples of 247 patients with T2DM and 887 normal subjects included in the AS–AS cohort of KoGES and identified potential DNA methylation sites, regions, and genes associated with diabetes in the Korean population. In previous cohort studies, several diabetes- or diabetic marker-related DMPs have been reported using EWAS. In particular, the cg19693031 region located in the 3′UTR of *TXNIP* has been reported to be highly associated with diabetic markers [19,20,21]. Thioredoxin-interacting protein (TXNIP), encoded by *TXNIP*, plays a crucial role in cellular processes, including redox regulation, metabolism, and cell growth. TXNIP is associated with diabetes mellitus, specifically T2DM, and regulates diabetes-related cellular functions such as glucose homeostasis, insulin resistance, and pancreatic β cell function [22]. Intervention with TXNIP represents a new strategy for treating diabetes mellitus [23,24]. Our DMP analysis identified cg19693031 as the most significant DMP associated with diabetes, and cg26964062, located within *TXNIP*, was sequentially identified as an important CpG site. Collectively, hypomethylation of the two CpG sites in *TXNIP* identified through EWAS analysis suggests that it may play an important role in the pathogenesis of diabetes mellitus in the Korean population.

CpG sites located within *PDK4*, *ABCG1*, *NBPF20*, *UFM1*, *PFKFB2*, and *ARRDC4* were identified within the 10 top DMPs. Pyruvate dehydrogenase kinase 4, encoded by *PDK4*, plays an important role in glucose metabolism and oxidation of fatty acids, and its upregulation is a factor in developing diabetes [25,26]. Consistent with the results of our DMP analysis, the cg17075888 CpG site of *PDK4* has been found to be significantly hypomethylated in patients with T2DM (*n* = 1534) [27]. *ABCG1* is also related to insulin resistance [28], and the association of a related CpG site cg06500161 identified by DMP analysis with diabetic markers has been reported [29]. The relationship between cg16740586 (located within *ABCG1*) and diabetes has not yet been reported; however, this is a novel DMP associated with obesity [30]. A direct relationship between NBPF Member 20 and pathogenesis of diabetes has not yet been reported; however, hypomethylation in the diabetic group through EWAS analysis of cg26823705 located within *NBPF20* has been reported, which is consistent with our study [31]. *PFKFB2*, identified in this study of T2DM mechanisms using 1026 Qatar BioBank samples, showed causal associations with HbA1c. Analysis of 66 T2D-CpG associations and whole-genome SNP associations implicated *PFKFB2* in metabolic networks related to T2DM [32]. *UFM1*, identified in a study of MKR mice modeling T2DM, is associated with endoplasmic reticulum-associated degradation in islet dysfunction, revealing its potential role in T2DM development [33]. cg10217853, located within *ARRDC4*, has been identified as a novel CpG site in this study, and arrestin domain–containing protein 4 is associated with diabetes by regulating insulin resistance and lipid metabolism in β cells in response to glucose [28,34]. Collectively, the DMP analysis in this EWAS suggests that relationships may exist between genes related to the top DMPs with high significance and the pathogenesis of diabetes mellitus.

By analyzing DMRs between diabetic and healthy individuals, we identified five genes that were hypomethylated (*DIP2C*, *FLI90757*, *PRSS50*, and *TDRD9*) or hypermethylated (*PDE1C*) in the diabetic group compared to those in the normal group. Notably, the mRNA expression of *TDRD9* increases in the visceral adipose tissue of patients with T2DM [35]. The enzyme phosphodiesterase 1C is encoded by *PDE1C* and regulates the levels of cAMP and cGMP in cells. It is also involved in the molecular pathway that regulates insulin secretion from pancreatic β cells [36]. Collectively, the results of DMR analysis suggest the potential significance of the DMR-annotated genes in the context of diabetes, indicating their involvement in various cellular processes and relevance to diabetes-related mechanisms.

In conclusion, the DNA methylation markers found through EWAS in the AS–AS cohort of KoGES hold significant value for future research on diagnostic markers of diabetes and may indicate the pathogenesis of diabetes through changes in DNA methylation. There are several limitations of the present study. First, the findings are based on a single cohort using a case–control design, which lack replications from an independent cohort, and, therefore, the results of the current study are hypothesis-generating in nature, which need to be corroborated in other independent studies. Second, the study participants are of Korean ethnicity, and the results may not be generalizable to other ethnic populations. Third, additional analyses could be necessary for separate EWASs with FPG, HbA1c, and 2 h PG levels as outcome variables. We further suggest exploring different pathways associated with DNA methylation by performing EWAS analyses with individual glycemic markers and future research directions, including the construction of regression models and the application of machine learning algorithms to interpret feature importance. Integrating these advances into our study could provide comprehensive insights into the intricate relationships between DNA methylation patterns and glycemic markers, thereby increasing the robustness and applicability of our findings. In addition, SNPs are the most prevalent type of genetic variant, and multiple linked SNPs in the same chromosomal region can be phased to haplotypes [37]. Third-generation sequencing such as Oxford Nanopore Technologies (ONT) and Pacific Biosciences (PacBio) single-molecule real-time sequencing are major long-read sequencing technologies that can be applied to determine DNA sequence and to detect DNA methylation simultaneously. Such long reads could cover more than several kilobases, thus helping resolve haplotype phases in genomic regions with low contents of SNPs. Recently, a software program called NanoMethPhase has been developed that can perform DNA methylation phasing at Mb scale based on the long reads generated by nanopore sequencing technologies [38]. In the future, such third-generation sequencing technologies could be applied to perform methylome sequencing for identifying DMPs and DMRs with haplotype phase information, which can facilitate identification of genetically imprinted regions.

## Figures and Tables

**Table 1:** Comparisons of Clinical and Biochemical Parameters in the KoGES Cohort Participants with Different Glycemic Status.

KoGES Cohort (*n* = 1134)	Normal (*n* = 887)			T2DM (*n* = 247)			*p* Value ^a^
	Mean	SD	*n*	Mean	SD	*n*	
Sex (% male)	50.20		887	57.50		247	-
Age (years)	57.77	8.38	887	62.45	8.22	247	***
BMI (kg/m^2^)	23.64	2.77	887	25.31	2.79	247	***
Smoking habit (%)	37.80		887	47.30		247	-
Fasting glucose (mg/dL)	89.26	5.64	887	161.05	40.97	247	***
HbA1c (%)	5.35	0.21	887	7.56	1.05	247	***
2-h plasma glucose (mg/dL)	105.49	21.25	887	261.96	58.38	56	***
Fasting insulin (µIU/mL)	7.63	3.04	887	13.57	18.69	247	***
2-h plasma insulin (µIU/mL)	28.69	27.68	887	36.68	29.04	56	***
Newly detected DM (*n*, %)				215, 87.0		247	-
DM treatment (*n*, %)				191, 88.8		215	-
Oral DM medication (*n*, %)				180, 83.7		215	-
Insulin treatment, (*n*, %)				20, 9.3		215	-
BUN (mg/dL)	15.26	3.94	887	16.43	5.89	247	*
Creatinine (mg/dL)	0.93	0.15	887	0.99	0.38	247	*
AST(SGOT) (IU/L)	25.28	9.19	887	26.47	11.88	247	n.s.
ALT(SGPT) (IU/L)	22.16	12.60	886	27.90	16.19	247	***
Total Cholesterol (mg/dL)	191.87	32.92	887	188.01	36.08	247	n.s.
HDL-Cholesterol (mg/dL)	44.68	11.50	887	39.51	8.56	247	***
Triglyceride (mg/dL)	123.48	73.37	887	181.91	112.83	247	***
hs-CRP (mg/L)	1.33	3.81	887	2.12	4.96	247	n.s.
W.B.C. blood (Thous/uL)	5.22	1.35	887	6.13	1.59	247	***
R.B.C. blood (Mil/uL)	4.43	0.42	887	4.46	0.46	247	n.s.
Hemoglobin (Hb) (g/dL)	13.70	1.38	887	13.73	1.49	247	n.s.
Hematocrit (Hct) (%)	41.17	3.84	887	41.06	4.23	247	n.s.
Platelet (Thous/uL)	255.22	60.11	887	255.35	66.94	247	n.s.

^a^, *, *p* < 0.001; ***, *p* < 0.000001; n.s., not significant (*p* ≥ 0.05).

**Table 2:** Top 10 differentially methylated probes (DMPs).

Probe	Delta-Beta	*p*-Value	Adj. *p*-Value	CHR	MAPINFO	Gene	Feature	cgi	Methylation
cg19693031	−0.058	2.95 × 10^−49^	2.13 × 10^−43^	1	145441552	TXNIP	3′UTR	opensea	hypo
cg26974062	−0.022	2.76 × 10^−36^	9.97 × 10^−31^	1	145440734	TXNIP	Body	opensea	hypo
cg26823705	−0.029	3.22 × 10^−23^	5.82 × 10^−18^	1	145435523	NBPF20	Body	opensea	hypo
cg04816311	0.021	2.34 × 10^−20^	2.42 × 10^−15^	7	1066650	C7orf50	Body	shore	hyper
cg17075888	−0.033	2.52 × 10^−19^	2.03 × 10^−14^	7	95225339	PDK4	Body	shore	hypo
cg16740586	0.026	3.05 × 10^−18^	2.21 × 10^−13^	21	43655919	ABCG1	Body	shore	hyper
cg02841972	−0.021	1.90 × 10^−15^	1.06 × 10^−10^	2	10176151		IGR	opensea	hypo
cg19750657	0.025	4.10 × 10^−15^	1.98 × 10^−10^	13	38935967	UFM1	3′UTR	opensea	hyper
cg10217853	−0.037	3.99 × 10^−14^	1.70 × 10^−9^	15	98505199	ARRDC4	Body	shore	hypo
cg00683922	0.021	4.66 × 10^−14^	1.87 × 10^−9^	1	207242569	PFKFB2	Body	opensea	hyper

**Table 3:** Top 10 differentially methylated regions (DMRs).

	Chromosome	Start	End	Width	*p* Value	*p* Value Area	Gene
DMR_1	chr6	32063114	32065211	2097	0	0.00040626	TNXB
DMR_2	chr6	30038910	30039600	690	0.0002208	0.00106864	RNF39
DMR_3	chr5	135415693	135416613	920	0.0006271	0.002040131	MIR886
DMR_4	chr10	530635	531584	949	0.0011923	0.003170594	DIP2C
DMR_5	chr1	153599479	153600156	677	0.0018193	0.004265729	S100A13
DMR_6	chr6	29648161	29649024	863	0.002049	0.010306638	
DMR_7	chr6	33047944	33048879	935	0.0022698	0.005581658	HLA-DPB1
DMR_8	chr6	31691354	31692152	798	0.0024464	0.017168898	C6orf25
DMR_9	chr6	31275551	31275881	330	0.0026937	0.006924082	
DMR_10	chr16	875257	875626	369	0.0026672	0.037163952	

## Data Availability

The data presented in this study are available in the article.

## Supplementary Materials

The following supporting information can be downloaded at: https://www.mdpi.com/article/10.3390/genes14122207/s1, Figure S1: Filtration process of HM850k probes; Figure S2: Singular value decomposition (SVD) analysis before (A) and after (B) batch effect correction. The labels “PC-1” to “PC-20” correspond to the Principal Components showing the top components correlated with the detected covariates (age, sex, Array, Slide, and Sample_Group), with the number of PCs determined using Random Matrix Theory; Figure S3: Normal Q-Q plot. The *x*-axis displays the theoretical quantiles expected under a normal distribution, whereas the *y*-axis represents the observed quantiles from the dataset. A straight line indicates conformity to a normal distribution; Figure S4: Co-methylation pattern analysis for the DMR surrounding the PDE1C. The upper panel displays a regional Manhattan plot of the DMR surrounding the PDE1C gene. The *y*-axis represents −log_10_(*p*-value) for each CpG probe in the DMR, and the *x*-axis represents the genomic location of each CpG site. In the middle panel, annotation tracks for gene, CpG Island, Broad ChromHMM, Regulation, and SNPs are presented. Within the Broad ChromHMM track, the pink track corresponds to heterochromatin/low signal regions, and the sky blue and yellow tracks correspond to CTCF binding site and predicted weak enhancer/Cis-regulatory element within the regulation ENSEMBL track, respectively, as defined in the coMET user guide (https://www.bioconductor.org/packages/devel/bioc/vignettes/coMET/inst/doc/coMET.pdf; accessed on 30 November 2023). In the lower panel, the co-methylation pattern of CpG probes within the DMR is depicted, displaying a heatmap of Spearman correlation coefficients (*R*). In this heatmap, red indicates a positive correlation with higher “R” values, whereas blue indicates a negative correlation with lower “R” values; Figure S5: Co-methylation pattern analysis for the DMR surrounding the *TDRD9*. The upper panel displays a regional Manhattan plot of the DMR surrounding the *TDRD9* gene. The *y*-axis represents -log_10_(*p*-value) for each CpG probe in the DMR, and the *x*-axis represents the genomic location of each CpG site. In the middle panel, annotation tracks for gene, Broad ChromHMM, Regulation, and SNP are presented. Within the Broad ChromHMM track, the brown track corresponds to repressed regions, as defined in the coMET user guide (https://www.bioconductor.org/packages/devel/bioc/vignettes/coMET/inst/doc/coMET.pdf; accessed on 30 November 2023). In the lower panel, the co-methylation pattern of CpG probes within the DMR is depicted, displaying a heatmap of Spearman correlation coefficients (*R*). In this heatmap, red indicates a positive correlation with higher “*R*” values, whereas blue indicates a negative correlation with lower “*R*” values.
