# Supplementary material for: Epigenetic Profiling of Type 2 Diabetes Mellitus: An Epigenome-Wide Association Study of DNA Methylation in the Korean Genome and Epidemiology Study
Source: Genes (Basel). 2023 Dec 13;14(12):2207. doi: 10.3390/genes14122207 (PMC10743302; doi:10.3390/genes14122207)
Supplement: Supplementary file 1 [file genes-14-02207-s001.zip › genes-2719671-supplementary.pdf]

Epigenetic Profiling of Type 2 Diabetes Mellitus: An Epigenome-Wide Association Study of DNA Methylation in the Korean Genome and Epidemiology Study

Hyein Seo, Jae-Ho Park, Jin-Taek Hwang, Hyo-Kyoung Choi, Soo-Hyun Park and Jangho Lee *

Korea Food Research Institute, Wanju-gun 55365, Jeollabuk-do, Republic of Korea; hiseo@kfri.re.kr (H.S.); jaehopark@kfri.re.kr (J.-H.P.); jthwang@kfri.re.kr (J.-T.H.); chkyoung@kfri.re.kr (H.-K.C.); shpark0204@kfri.re.kr (S.-H.P.)

* Correspondence: jhlee@kfri.re.kr; Tel.: +82-63-219-9356

Supplementary Materials: Supplementary Figures S1, S2, S3, S4, and S5

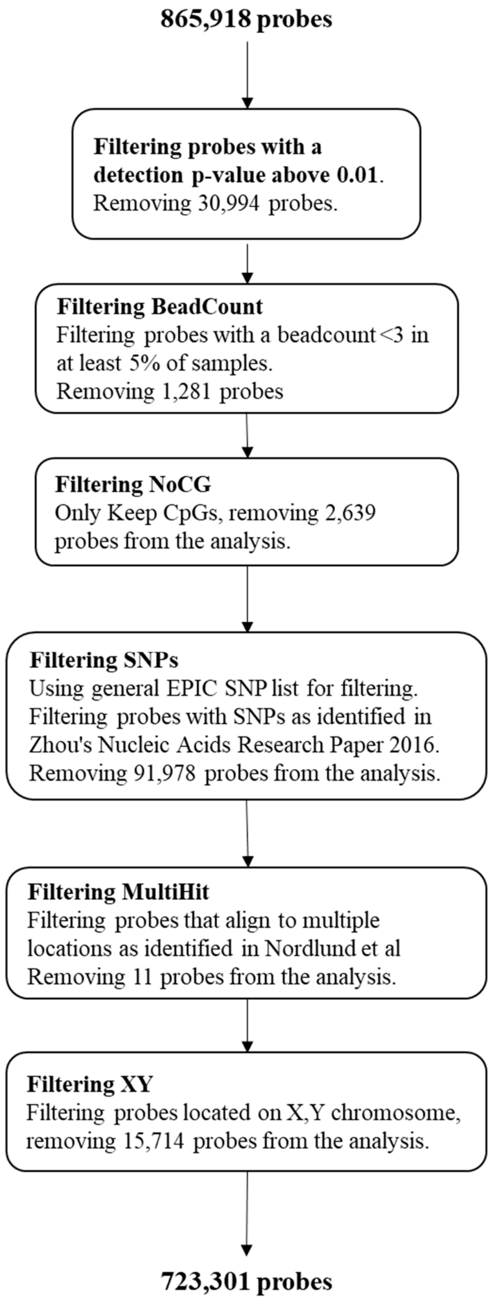

Supplementary Figure S1. Filtration process of HM850k probes.

A

B

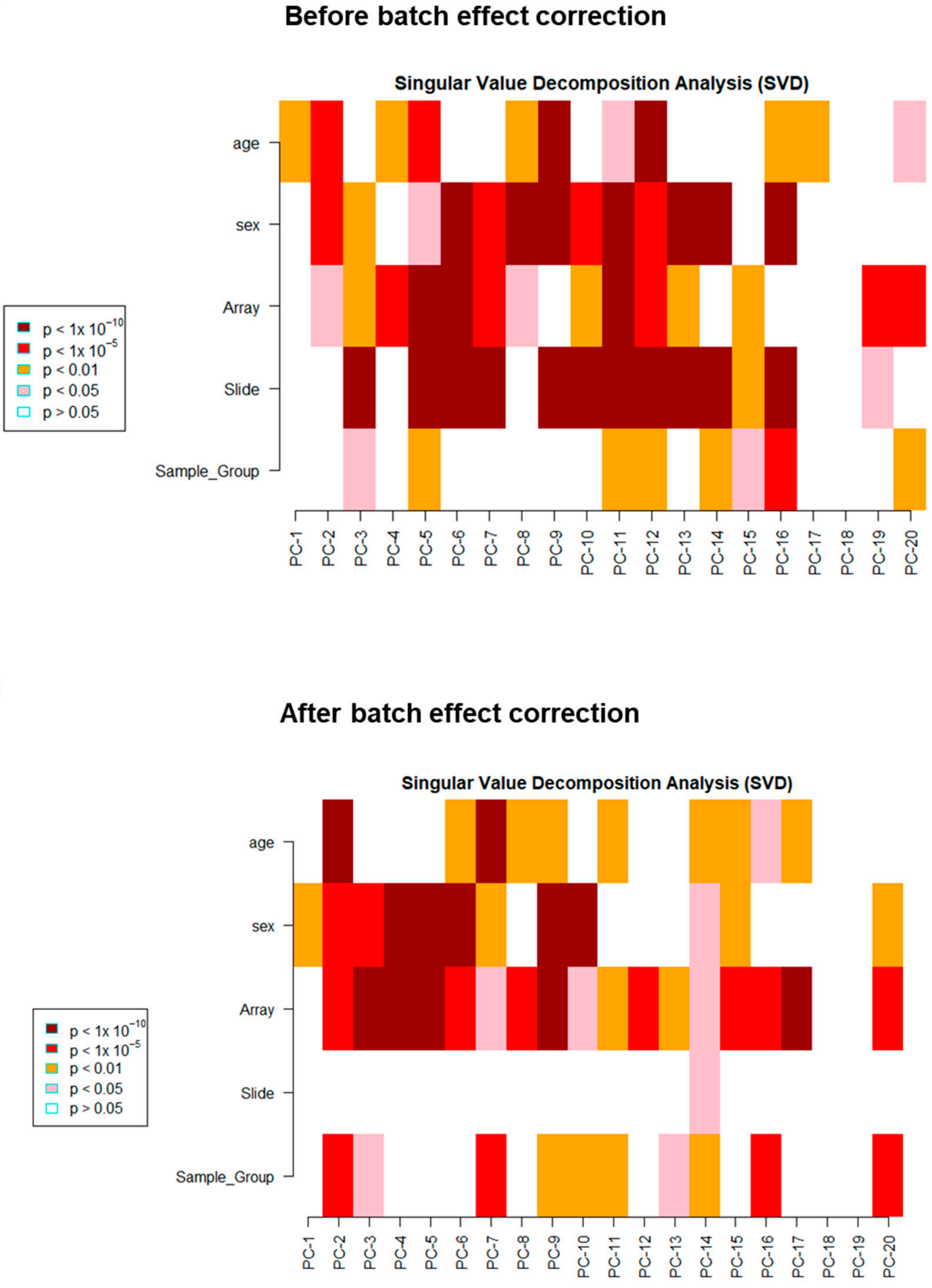

Supplementary Figure S2. Singular value decomposition (SVD) analysis before (A) and after (B) batch effect correction. The labels "PC-1" to "PC-20" correspond to the Principal Components (PCs) showing the top PCs correlated with the detected covariates (age, sex, Array, Slide, and Sample_Group), with the number of PCs determined using Random Matrix Theory.

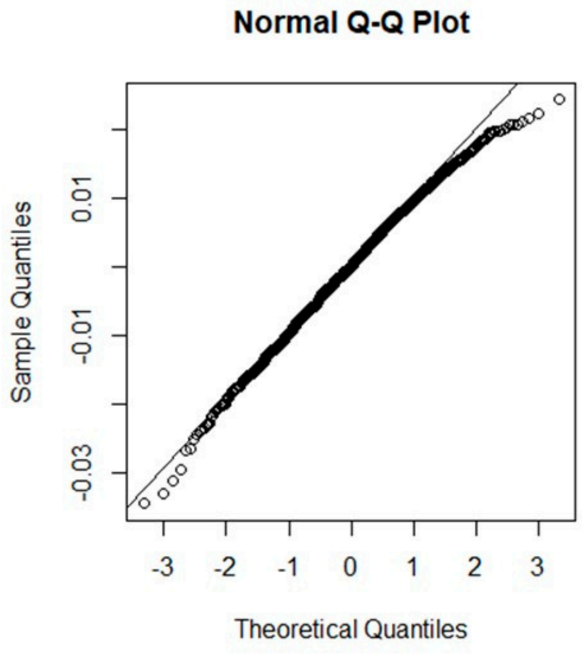

Supplementary Figure S3. Normal Q-Q plot. The x-axis displays the theoretical quantiles expected under a normal distribution, while the y-axis represents the observed quantiles from the dataset. A straight line indicates conformity to a normal distribution.

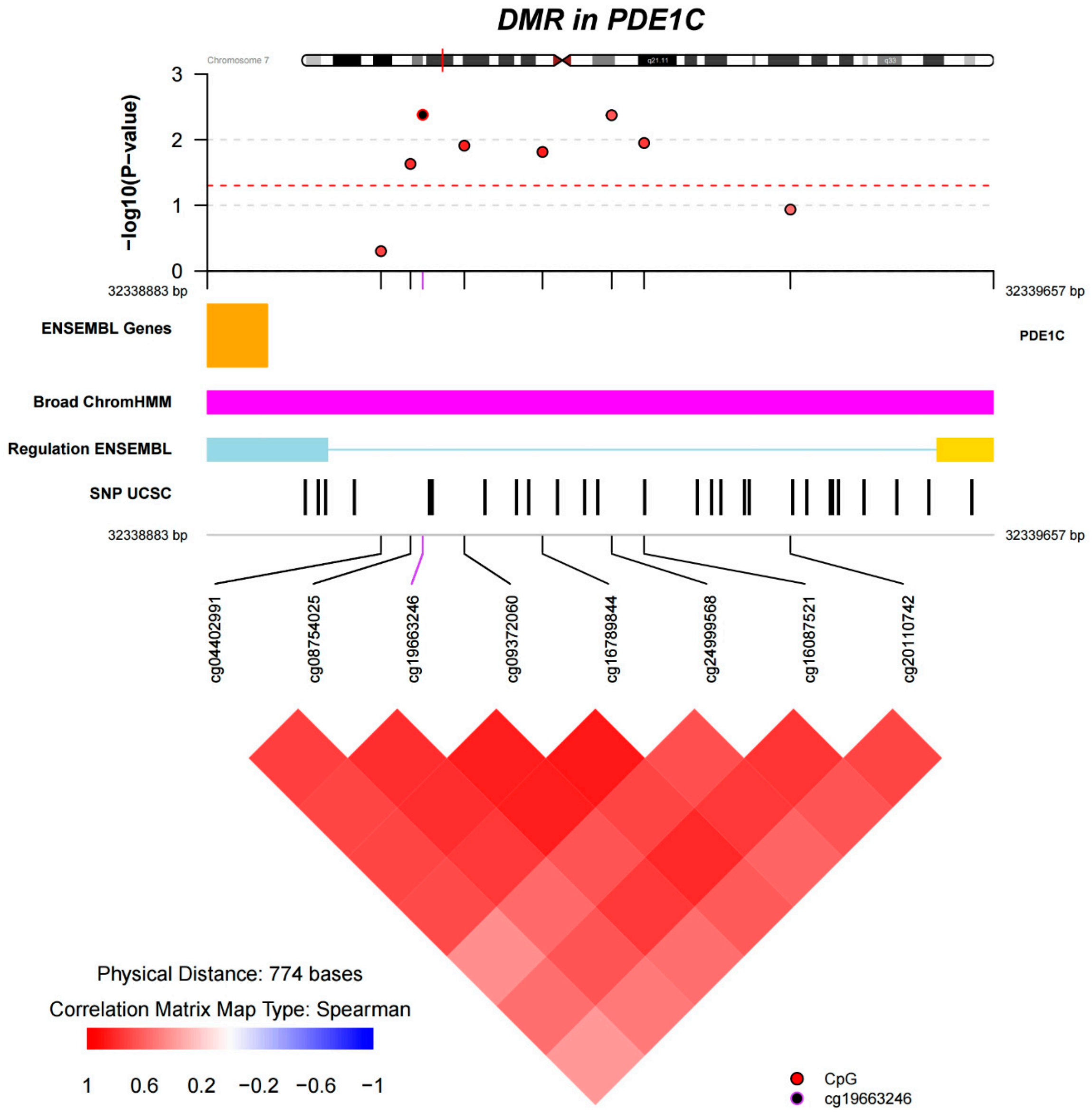

Supplementary Figure S4. Co-methylation pattern analysis for the DMR surrounding the *PDE1C*. The upper panel displays a regional Manhattan plot of the DMR surrounding the *PDE1C* gene. The y-axis represents -$\log_{10}$(p-value) for each CpG probe in the DMR, and the X-axis represents the genomic location of each CpG site. In the middle panel, annotation tracks for gene, Broad ChromHMM, Regulation, and SNP are presented. Within the Broad ChromHMM track, pink track corresponds to heterochromatin/low signal regions, and within the regulation ENSEMBL track, sky blue and yellow tracks correspond to CTCF binding site and predicted weak enhancer/*Cis*-regulatory element, respectively, as defined in the coMET user guide

(https://www.bioconductor.org/packages/devel/bioc/vignettes/coMET/inst/doc/coMET.pdf; accessed on 30 November 2023). In the lower panel, the co-methylation pattern of CpG probes within the DMR is depicted, displaying a heatmap of Spearman correlation coefficients (*R*). In this heatmap, red indicates a positive correlation with higher '*R*' values, while blue indicates a negative correlation with lower '*R*' values.

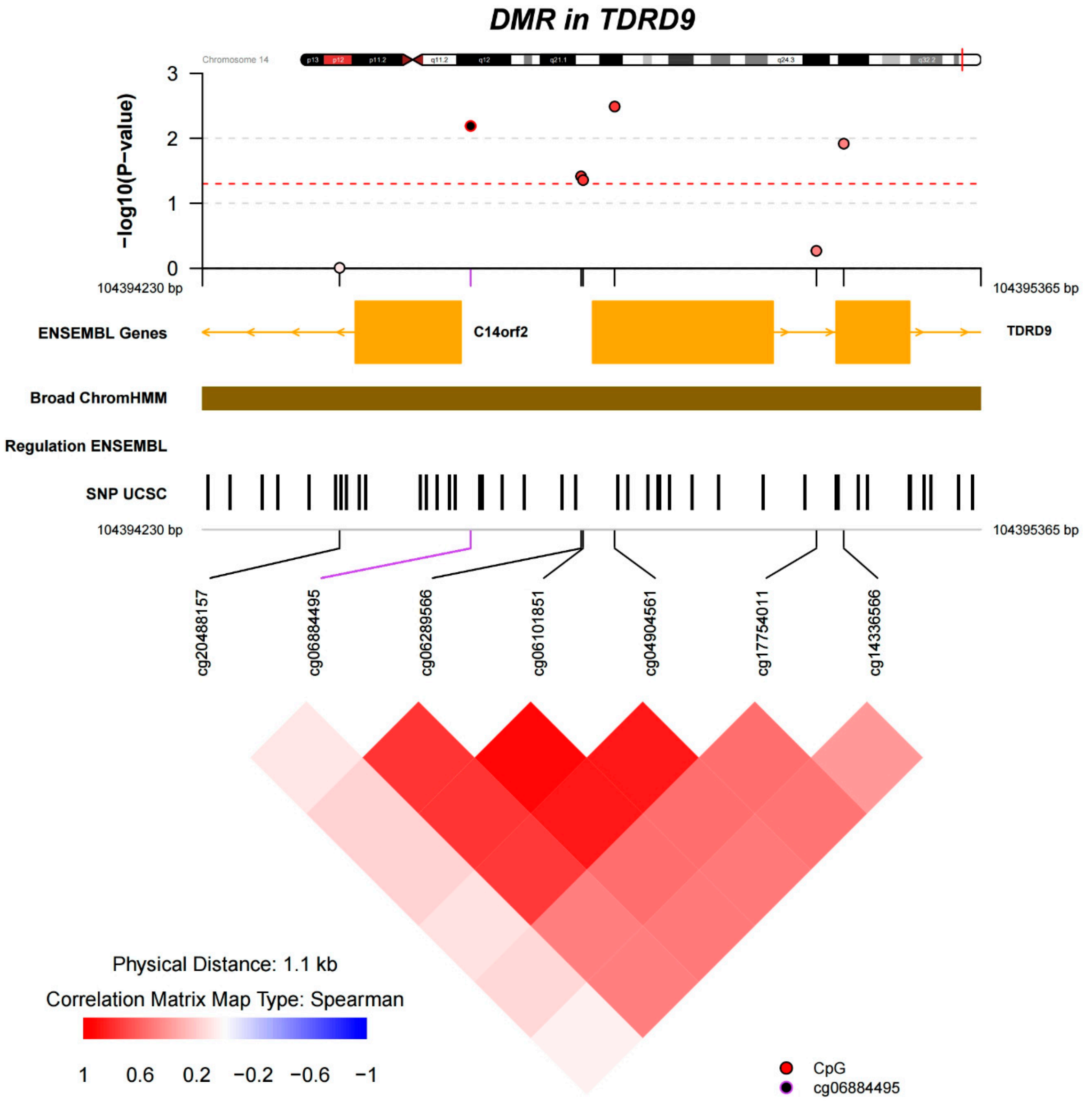

Supplementary Figure S5. Co-methylation pattern analysis for the DMR surrounding the *TDRD9*. The upper panel displays a regional Manhattan plot of the DMR surrounding the *TDRD9* gene. The y-axis represents -$\log_{10}$(p-value) for each CpG probe in the DMR, and the X-axis represents the genomic location of each CpG site. In the middle panel, annotation tracks for gene, Broad ChromHMM, Regulation, and SNP are presented. Within the Broad ChromHMM track, brown track corresponds to repressed regions, as defined in the coMET user guide (https://www.bioconductor.org/packages/devel/bioc/vignettes/coMET/inst/doc/coMET.pdf; accessed on 30 November 2023). In the lower panel, the co-methylation pattern of CpG probes within the

DMR is depicted, displaying a heatmap of Spearman correlation coefficients (*R*). In this heatmap, red indicates a positive correlation with higher '*R*' values, while blue indicates a negative correlation with lower '*R*' values.
